# Printing Formation of Flexible (001)-Oriented PZT Films on Plastic Substrates

**DOI:** 10.3390/ma16052116

**Published:** 2023-03-06

**Authors:** Tomohiko Nakajima, Yuuki Kitanaka

**Affiliations:** Advanced Manufacturing Research Institute, National Institute of Advanced Industrial Science and Technology, Ibaraki 305-8565, Japan

**Keywords:** PZT energy harvesters, flexible materials, laser processing, photo-assisted chemical solution deposition, photocrystallization, perovskite oxides, wearable devices, orientation growth

## Abstract

High-quality, uniaxially oriented, and flexible PbZr_0.52_Ti_0.48_O_3_ (PZT) films were fabricated on flexible RbLaNb_2_O_7_/BaTiO_3_ (RLNO/BTO)-coated polyimide (PI) substrates. All layers were fabricated by a photo-assisted chemical solution deposition (PCSD) process using KrF laser irradiation for photocrystallization of the printed precursors. The Dion–Jacobson perovskite RLNO thin films on flexible PI sheets were employed as seed layers for the uniaxially oriented growth of PZT films. To obtain the uniaxially oriented RLNO seed layer, a BTO nanoparticle-dispersion interlayer was fabricated to avoid PI substrate surface damage under excess photothermal heating, and the RLNO has been orientedly grown only at around 40 mJ·cm^−2^ at 300 °C. The prepared RLNO seed layer on the BTO/PI substrate showed very high (010)-oriented growth with a very high Lotgering factor (*F*(010) = 1.0). By using the flexible (010)-oriented RLNO film on BTO/PI, PZT film crystal growth was possible via KrF laser irradiation of a sol–gel-derived precursor film at 50 mJ·cm^−2^ at 300 °C. The obtained PZT film showed highly (001)-oriented growth on the flexible plastic substrates with *F*(001) = 0.92 without any micro-cracks. The RLNO was only uniaxial-oriented grown at the top part of the RLNO amorphous precursor layer. The oriented grown and amorphous phases of RLNO would have two important roles for this multilayered film formation: (1) triggering orientation growth of the PZT film at the top and (2) the stress relaxation of the underneath BTO layer to suppress the micro-crack formation. This is the first time that PZT films have been crystallized directly on flexible substrates. The combined processes of photocrystallization and chemical solution deposition are a cost-effective and highly on-demand process for the fabrication of flexible devices.

## 1. Introduction

Flexible materials have recently become significant in the development of next-generation electronic devices [1]. Electronic device manufacturing has completely changed in the last half decade and many devices are now designed with a high shape degree-of-freedom through recent manufacturing process innovations. Wearable devices are one of the most attractive research areas and are mainly used for healthcare management to extend life expectancy. Such wearable devices acting as sensor-integrated systems are notable for their real-time monitoring of patients for medical care [2,3,4,5,6,7,8,9]. For this new demand, efforts have been made to convert various device components, such as sensors and passive parts, into flexible forms [10,11,12,13,14,15,16]. Power source development has been a pressing issue in realizing such flexible devices. In particular, flexible self-powered energy harvesters using piezoelectric elements are expected to be good power source candidates. For piezoelectric power generation, piezoelectric films must be prepared on flexible substrates at low temperatures. Lead zirconate titanate (PZT) [17,18], a typical piezoelectric material, prepared on flexible plastic substrates as self-powered sensing applications have been reported [19,20,21,22]. The transferring laser lift-off method, wherein an epitaxial PZT film is grown on a single crystal substrate using pulsed laser deposition, is currently a very strong tool for fabricating flexible PZT films on plastic substrates [22]. While high crystal quality PZT films have been fabricated using this process, it still needs to be drastically improved in terms of cost for industrial applications.

To achieve low-cost and future high-mix low-volume production objectives [23,24], we have employed a photo-assisted chemical solution deposition (PCSD) process [25,26,27,28,29,30,31] for PZT fabrication. The chemical solution deposition (CSD) process is one of very simple printing techniques and enables on-demand fabrication, in contrast to conventional methods like the transferring laser lift-off method. Printing formation will be integrated with inkjet printing and 3D printer technology, and is expected to become an important technology for the formation of new devices, such as 3D electronics, in the future [32,33,34,35]. The PCSD process based on printing technology can be used to fabricate oxide thin films at low temperatures for commonly used substrates that cannot withstand high temperatures (*T* > 400 °C) in air. During the PCSD process, oxide films are crystallized through excimer laser irradiation instead of high-temperature furnace heating as is used in the CSD process. The photothermal gradient heating near the thin film surface is realized by a shallow penetration depth of the excimer laser with ultraviolet wavelength for oxide films in the PCSD process. We have previously fabricated uniaxially oriented Dion–Jacobson perovskite (DJP) RbLaNb_2_O_7_ (RLNO) [36,37] thin films on amorphous glass substrates by PCSD to create oriented-growth seed layers for other perovskite oxide films [38,39]. We conceived that a combination of some anisotropic structural properties of DJP and the gradient temperature distribution realized in the PCSD process has the potential for anisotropic crystal growth, leading to uniaxial-oriented film growth. Here, we fabricate oriented RLNO thin films on flexible polyimide (PI) substrates and high-quality uniaxially oriented PZT films on the RLNO seed layer. The whole multilayered flexible PZT film structure is prepared by printing-based methods; therefore, the PCSD process is expected to be an excellent option for the fabrication of PZT energy harvesters for industrial applications.

## 2. Materials and Methods

An interlayer of BaTiO_3_ (BTO) was prepared directly onto PI substrates with a thickness of 75 μm (Kapton 300H, Du Pont-Toray, Tokyo, Japan) by the PCSD process. First, 1.0 mL of BTO nanoparticle dispersion (BtMin, Nyacol, Ashland, MA, USA), 1.0 mL of 0.5 M Ba metalorganic solution (SYM-BA05, Symetrix, Mount Lake Terrace, WA, USA), and 1.0 mL of 0.5 M Ti metalorganic solution (SYM-TI05, Symetrix) were mixed by ultrasonication. The obtained BTO nanoparticle dispersion ink was spin-coated onto the PI substrates at 4000 rpm for 10 s. The coated BTO precursor films were dried at 300 °C in air for 10 min to decompose the organic components of the films. After spin coating and preheating, the films were irradiated with a KrF laser (Lambda Physik Compex110, Coherent, Dieburg, Germany) at a fluence of 40 mJ/cm^2^ and pulse duration of 26 ns for 2000 pulses at 300 °C in air. The coating, preheating, and laser irradiation steps were repeated four times to increase film thickness.

The DJP RLNO seed layers were also fabricated using the PCSD process. The starting solutions for the RLNO films were prepared by mixing 2-ethylhexanoate solutions of the constituent metals—Rb (SYM-RB03, Symetrix), La (SYM-LA01, Symetrix), and Nb (niobium 2-ethylhexanoate, Gelest, Morrisville, PA, USA)—and diluted with toluene to obtain the required concentration and viscosity for spin coating. The Rb:La:Nb molar ratio in the coating solution was 1.0:1.0:2.0. This solution was spin-coated onto the PI substrates with and without BTO interlayers. The coated RLNO precursor films were preheated at 300 °C in air for 10 min to decompose the organic components of the films, and the coating and preheating process was repeated four times. After spin coating and preheating, the films were irradiated with a KrF laser at a fluence of 35–55 mJ/cm^2^ for 7500 pulses at 300 °C in air. 

**Figure 1:**
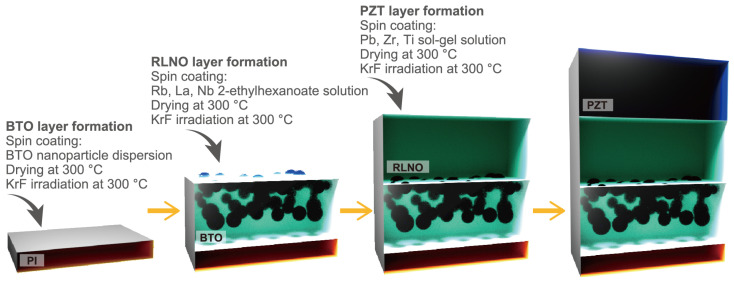
Flow chart of fabrication procedure for the PZT/RLNO/BTO/PI layer structure.

The obtained RLNO thin films were used as DJP seed layers for PZT film preparation. The PZT thin films on PI substrates with and without the DJP seed layer were prepared by the PCSD process. A starting sol–gel solution with lead, zirconium, and titanium (Pb:Zr:Ti = 1.10:0.52:0.48, PZT-N, Mitsubishi Materials, Tokyo, Japan) was spin-coated onto the substrates at 3000 rpm for 10 s. The coated films were dried at 200 °C in air for 10 min. The preheated films were irradiated with the KrF laser at a fluence of 50 mJ/cm^2^ for 5000 pulses at 300 °C in air. The coating, preheating, and laser irradiation processes were repeated ten times to increase the film thickness. The fabrication procedure is briefly summarized in Figure 1.

The crystal structure and orientation properties of the obtained films were studied by X-ray diffraction (XRD) measurements (Rigaku, SmartLab, Tokyo, Japan). 2θ-β maps (β is the direction along the Debye rings) were obtained with a two-dimensional (2D) pixel area detector (Dectris, PILATUS 100K, Baden, Switzerland) and a collimator with a 200-μm diameter. The distance between the detector and the samples was fixed at 120 mm. The degree of crystal orientation was evaluated in terms of the Lotgering factor, *F*, which is calculated from the following equation [40]:

(1) $$F\left(hkl\right)=\left(P-P_{0}\right)/\left(1-P_{0}\right)$$

where *P*_0_ = Σ *I*_0_(*hkl*)/Σ *I*_0_(*HKL*) and *P* = Σ *I*(*hkl*)/Σ *I*(*HKL*). In these expressions, *I*_0_ and *I* are the intensities of each diffraction peak in the XRD patterns as presented from the ICSD database and experimental data, respectively. The crystal growth process and crystallinity of the thin films were observed by cross-sectional transmission electron microscopy (XTEM) analysis using an H-9000NAR (Hitachi High-Tech, Tokyo, Japan) instrument operating at 300 kV. 

Temperature variations during laser irradiation were simulated by the heat diffusion equation simplified for one-dimensional heat flow:

(2) $$\rho_{m}C\frac{\partial T}{\partial t}=\kappa \frac{\partial^{2}T}{\partial z^{2}}+\alpha I\left(z,t\right)$$

where *T* is the temperature function at time *t* and depth *z*, *ρ*_m_ is the mass density, *C* is the specific heat capacity, *α* is the optical absorption coefficient, *κ* is the thermal conductivity, and *I*(*z*, *t*) is the laser power density.

## 3. Results and Discussion

**Figure 2:**
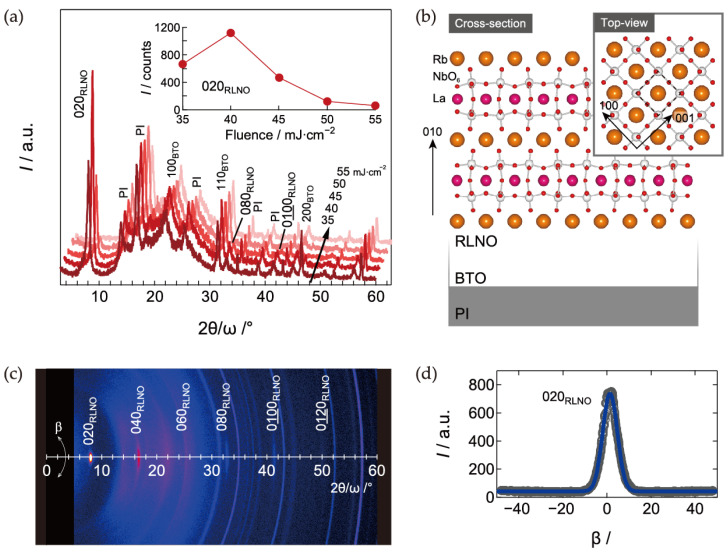
(**a**) XRD pattern irradiated laser fluence dependence for RLNO films on BTO/PI substrates prepared by the PCSD process. (**b**) Schematic layers and crystal structure of RLNO. The dotted line in the inset represents the simple perovskite unit cell. (**c**) XRD 2θ-β map of an RLNO film on BTO/PI prepared at a fluence of 40 mJ·cm^−2^. (**d**) β-scan for the 020 reflection. The blue line indicates the fitting for full width at half maximum (FWHM) evaluation.

Figure 2a shows the XRD patterns for the RLNO films on PI substrates with BTO interlayers (RLNO/BTO/PI) prepared by the PCSD process. The 2θ/ω XRD scans show the crystallization of RLNO, and all reflections related to the RLNO were assigned to (0*k*0) indices without any other orientations. The Lotgering factor *F*(010) was 1.0 and this orientation simply indicates Dion–Jacobson layer stacking-type ordering. The simple perovskite lattice unit appears at the surface (Figure 2b). The crystallinity increased at a fluence of 40 mJ·cm^−2^ and decreased with increasing laser fluence above 45 mJ·cm^−2^. An XRD 2θ-β map of the RLNO film on BTO/PI prepared at a fluence of 40 mJ·cm^−2^ is shown in Figure 2c. The Bragg reflections corresponding to (0*k*0) indices appear as a spot, and the full width at half maximum (FWHM) of the (020) reflection was 5.51° (Figure 2d). This value is larger than that for RLNO fabricated on flat glass substrates (FWHM = 3.37°) [38]. This narrow FWHM for the RLNO films on flexible substrates indicates that the first nucleation of RLNO at the precursor surface starts with a highly uniaxially oriented state, which originated from a strong two-dimensional feature of the DJP layered structure. The nucleation of the layered structure—that is, (010)-orientation of RLNO—favorably reduces the surface energy at the flat precursor surface [38,39].

**Figure 3:**
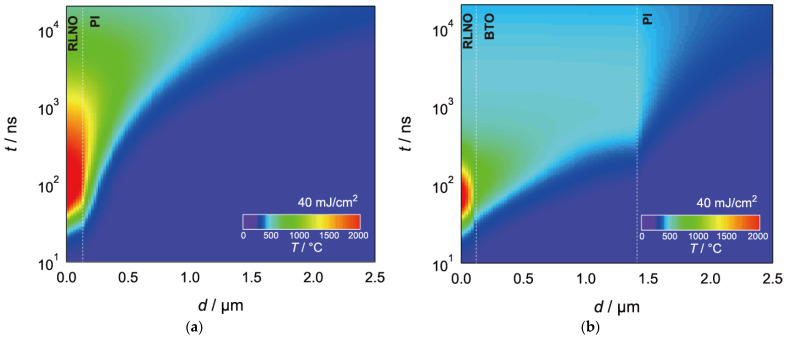
Numerically simulated temperature maps with depth (*d*) and time (*t*) for the amorphous precursor RLNO thin film on the PI substrate irradiated by a 40-mJ·cm^−2^ KrF laser pulse (**a**) without and (**b**) with the BTO interlayer.

While we have successfully obtained flexible RLNO films, oriented growth of RLNO on the flexible substrates was very difficult compared to that grown on rigid flat substrates, such as glass, because of the management of the instantaneous photothermal heating effect under pulsed laser irradiation. The PI substrate has a much smaller thermal conductivity (*κ* = 0.0012 W·cm^−1^K^−1^) than that of silica glass (*κ* = 0.015 W·cm^−1^K^−1^). This small thermal conductivity causes a reduction in heat diffusion from the precursor films to the substrates. Figure 3a shows the results of theoretical temperature simulations for an amorphous RLNO precursor film without the BTO interlayer under 26 ns irradiation by 40 mJ·cm^−2^ KrF laser pulses. The maximum temperature of the top surface reaches 2105 °C 48 ns after the incident pulse, and the high temperature, above 1000 °C, is maintained for 2.8 μs. As a result, the excess photothermal heating damaged the PI substrate and the precursor RLNO film vanished by laser ablation. 

On the other hand, the introduction of the BTO interlayer with relatively large thermal conductivity (*κ* = 0.05 W·cm^−1^K^−1^) maintained the RLNO precursor film without any layer-structure damage. The BTO interlayer realizes heat management under the laser pulse. Figure 3b shows temperature simulations for the amorphous RLNO precursor film with a 1.3 μm BTO interlayer under 26 ns of 40 mJ·cm^−2^ KrF laser pulses. The insertion of the BTO layer between the amorphous RLNO and the PI substrate reduces the high temperature region above 1000 °C, and the temperature region above 1000 °C is reduced after only 130 ns. The calculated maximum temperature is suppressed to 1710 °C, which is comparable to the case of 56 mJ·cm^−2^ irradiation of the RLNO precursor on a glass substrate [38]. This strongly suggests the importance of BTO layer insertion for properly controlling thermal properties underneath the RLNO precursor.

**Figure 4:**
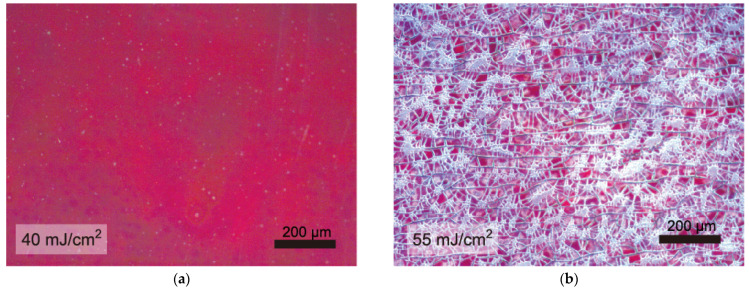
Optical microscope images for RLNO/BTO/PI prepared by KrF laser irradiation at (**a**) 40 mJ·cm^−2^ and (**b**) 55 mJ·cm^−2^.

Nevertheless, the laser fluence should be precisely controlled, even with the BTO interlayer. The crystallized RLNO film on the BTO/PI substrate maintained its thin-film form without any cracks. However, the dense-film form of RLNO was damaged when the fluence increased to 55 mJ·cm^−2^ (Figure 4). The optical microscope image for the sample after laser irradiation at 55 mJ·cm^−2^ for 7500 pulses showed reticulated cracks across the entire sample. This type of mud-crack pattern is because of the shrinkage stress relaxation and is usually observed in CSD-based films during the densification process, which further led to crystallization [41,42,43,44]. The internal stress (*σ*_ther_) at the interface due to the thermal expansion mismatch between the RLNO and BTO films is calculated as follows [45]:

(3) $$\sigma_{\mathrm{ther}}=\frac{Y}{1-\sigma }\Delta \theta_{t}\Delta T$$

where *Y* is Young’s modulus of the film, *σ* is its Poisson’s ratio, Δ*θ*_t_ is the difference in linear thermal expansion coefficients for the film and substrate, and Δ*T* is the temperature variation during laser irradiation. The Δ*θ*_t_ values between the amorphous precursor and the nanoparticle-derived polycrystalline layer are not similar at the interface. This means that *σ*_ther_ cannot be ignored during laser irradiation [14]. *σ*_ther_ is calculated as a linear increase in Δ*T* (Equation (3)) and, thus, the threshold for film degradation can be roughly evaluated from photothermal temperature variations.

**Figure 5:**
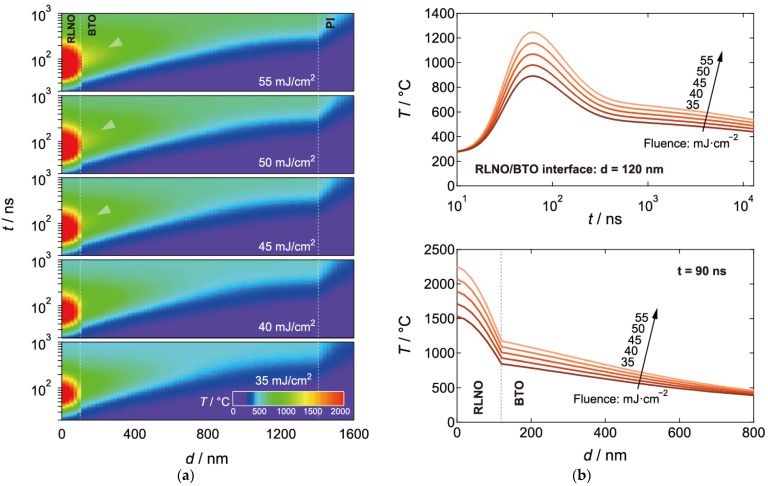
(**a**) Numerically simulated temperature maps across depth (*d*) and time (*t*) for the amorphous precursor RLNO thin film on a BTO/PI substrate irradiated at a KrF laser fluence of 35–55 mJ·cm^−2^. (**b**) Time dependence at the interface between RLNO and BTO (*d* = 120 nm) and depth dependence (*t* = 90 ns) of simulated temperature for the RLNO/BTO layers irradiated at a KrF laser fluence of 35–55 mJ·cm^−2^.

Figure 5a shows simulated temperature maps for the amorphous RLNO precursor film with BTO interlayer under KrF laser pulse at 35–55 mJ·cm^−2^. The photothermal heating effect on the maximum temperature increases with increasing fluence, and the high temperature region above 1500 °C extends more than 200 ns from the incident pulse above 45 mJ·cm^−2^. The maximum temperature at the surface also increased up to 1891 °C and almost linearly increases with laser fluence, reaching 2251 °C at 55 mJ·cm^−2^, as shown in Figure 5b. The pulse shape of temperature related to the decay curve tends to be decreasing below 800 °C. This leads to growing heat accumulation in the RLNO layer, resulting in a high temperature region above 1000 °C at the BTO layer via the interface (yellow parts indicated by white arrows in Figure 5a). While the temperature profiles at the interface between the RLNO and BTO layers vary with fluence, they are not largely affected at the PI substrate surface. 

The temperature decay at each fluence is almost finished at a depth of 1.4 μm (Figure 5b). Thus, the internal stress of RLNO at the interface with the BTO interlayer due to heat shock and originating from strong instantaneous heating above 45 mJ·cm^−2^ could be causing the cracks observed in the RLNO films. To employ the RLNO films as seed layers for other perovskites, the laser fluence should be kept around 40 mJ·cm^−2^ to combine good surface crystallinity and flatness while avoiding cracks. For the oriented growth of PZT films in this study, we used the uniaxial (010)-oriented RLNO films prepared at 40 mJ·cm^−2^ as seed layers.

**Figure 6:**
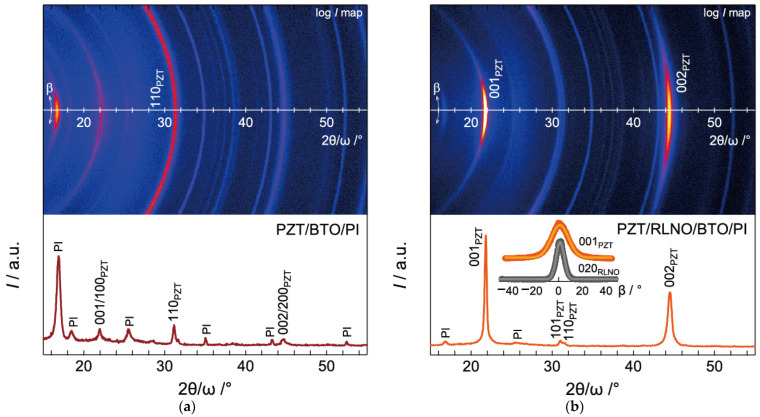
XRD patterns and 2θ-β maps for PZT films prepared by the PCSD process at 50 mJ·cm^−2^ on BTO/PI substrates (**a**) without and (**b**) with (010)-oriented RLNO seed layers. The inset shows the β-scan for the 001 spot of the PZT and the 020 spot of the RLNO films.

Figure 6 shows the XRD 2θ-β maps and 2θ/ω scans of the PZT films prepared by the PCSD process at 50 mJ·cm^−2^ on BTO/PI without and with (010)-oriented RLNO seed layers. In the 2θ/ω scan for the PZT/BTO/PI, no preferential orientation was confirmed, whereas the PZT was crystallized by the PCSD process as a single phase without impurities (Figure 6a). The Debye rings for the 100/001 and 110 reflections are observed in the 2θ-β map. On the contrary, the XRD 2θ/ω scans for the PZT film prepared by PCSD on the RLNO/BTO/PI show strong uniaxial orientation. The intense 001 and 002 reflections and small hump peaks related to 101/110 reflections were observed, as shown in Figure 6b. The Lotgering factor *F*(001) of the PZT was very high (0.92). This strong (001)-orientation originates from simple perovskite lattices exposed at the topmost surface by the (010)-orientation of RLNO. Preferential cube-on-cube growth is a significant feature of photocrystallization and such growth proceeds for the PZT on the oriented RLNO seed layer.

In this system, the lattice mismatch between the PZT and RLNO is 4.02% (*a* = 0.3885 nm in RLNO [36] and *a* = 0.404 nm in PZT [17]), which is slightly larger than that previously reported for SrTiO_3_ (0.53%) and LaNiO_3_ (1.15%) [39]. The Lotgering factors (*F*(001)) for SrTiO_3_ and LaNiO_3_ on the RLNO seed layer were 0.983 and 0.971, respectively. Since a large lattice mismatch between film and substrate directly connects to the deterioration of orientation growth, the slight reduction of orientation degree in the PZT on RLNO is derived from their large lattice mismatch compared to the SrTiO_3_ and LaNiO_3_. In the 2θ-β map for the PZT on the RLNO seed layer, the spot-like reflections assigned to (00*l*) were observed (Figure 6b). The β scan of Bragg reflections corresponding to the (001) reflection maintained its peak shape and the FWHM was evaluated to be 12.14°.

**Figure 7:**
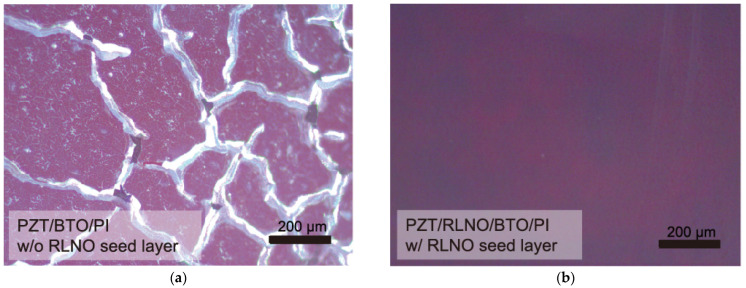
Optical microscope images for the PZT films prepared by the PCSD process at 50 mJ·cm^−2^ (**a**) without and (**b**) (010)-oriented RLNO seed layers.

**Figure 8:**
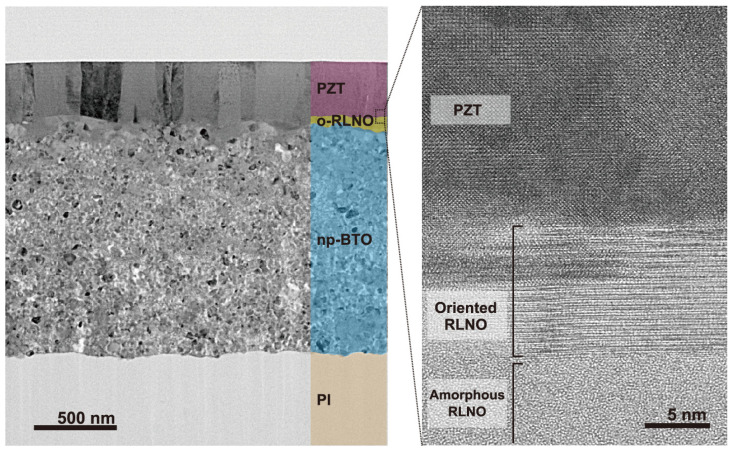
XTEM image of the PZT/RLNO/BTO/PI and high-resolution lattice image at the interface between PZT and the (010)-oriented RLNO film surface. o- and np- represent “oriented” and “nanoparticle-derived”, respectively.

The other role of the RLNO seed layers is to form highly crystalline and oriented PZT films. Figure 7 shows optical microscope images for PZT films prepared by the PCSD process at 50 mJ·cm^−2^ without and with the (010)-oriented RLNO film. The film appearances are completely different. The PZT film crystallized on the RLNO seed layer did not indicate the generation of any film-surface deterioration while that prepared without the seed layer showed cracks. The XTEM images were collected to confirm the crystal quality of the PZT film obtained with the RLNO seed layer, as shown in Figure 8.

**Figure 9:**
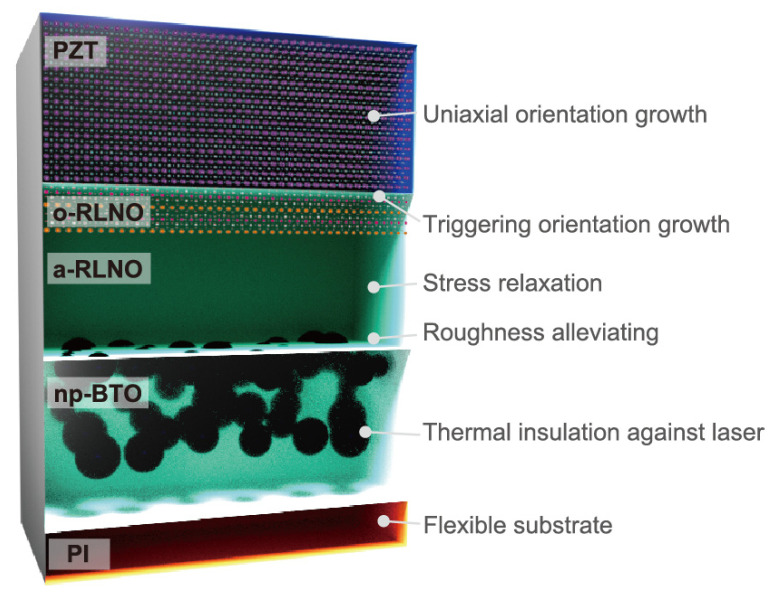
Schematic illustration of the PZT/RLNO/BTO/PI layer structure with the role of each layer. o-, a-, and np- represent “oriented”, “amorphous”, and “nanoparticle-derived”, respectively.

The XTEM image for the PZT/RLNO/BTO/PI shows a clear layered structure of the PZT, RLNO, and BTO phases, and each thickness was confirmed to be 500 nm (PZT), 100 nm (RLNO), and 1.3 μm (BTO), respectively. The BTO layer had a relatively large surface roughness derived from the original particulate shape of the BTO nanoparticles. The RLNO layer was deposited on this BTO layer to alleviate the granular roughness. It is noteworthy that the RLNO layer showed a highly (010)-oriented region only at the top part, while the bottom part, making contact with the BTO layer, remained amorphous. It is confirmed that the (010)-oriented seed of RLNO at the interface with the PZT layer encouraged cube-on-cube PZT film growth. Underneath this PZT layer, the amorphous region could assume a role of stress relaxation from the BTO nanoparticle layer and simply improve roughness to avoid crack formation. Although the interface at the RLNO and PZT layers still has some roughness, it was completely resolved at the topmost surface of the PZT layer. The PZT layer exhibited columnar growth on the oriented RLNO layer via an epitaxial relationship. Therefore, the large FWHM of the β scan (Figure 6b) could originate from the slight tilt of the RLNO layer surface due to roughness while the crystal quality of the PZT itself is high based on the XTEM results. Thus, we have achieved the fabrication of flexible uniaxial (001)-oriented PZT films on plastic substrates by a printing method based on photocrystallization. Although it involves orientation control of ceramic films prepared by printing-based methods only on ceramic substrates with sufficient resistance against heat treatments [31,38,46], this is the first case to enable orientation growth on plastic substrates. We confirmed that precise control of two different interlayers—BTO and RLNO—fulfills an essential role in obtaining high-quality PZT crystallization under KrF laser irradiation, as summarized in Figure 9. This “print-on-demand” technique for the fabrication of uniaxially oriented PZT films will enable the facile fabrication of flexible piezoelectric devices on plastic substrates. 

## 4. Conclusions

We investigated a new fabrication process for high-quality, uniaxial-oriented growth of flexible PZT films. The PZT films were fabricated on flexible RLNO/BTO/PI-layered substrates. All layers were fabricated by the PCSD process using KrF laser irradiation for photocrystallization of the printed precursors. DJP RLNO thin films on flexible PI sheets were employed as seed layers for the uniaxially oriented growth of PZT films. To obtain uniaxially oriented RLNO seed layers, a BTO nanoparticle dispersion-deposited interlayer was fabricated to avoid PI substrate damage under excess photothermal heating, and the RLNO has been orientedly grown only at around 40 mJ·cm^−2^ at 300 °C. The prepared RLNO seed layer on the BTO/PI substrate showed very high (010)-oriented growth with *F*(010) = 1.0. Then, PZT films were fabricated by KrF laser irradiation of a sol-gel-derived precursor film at 50 mJ·cm^−2^ at 300 °C using the flexible (010)-oriented RLNO film on BTO/PI. The obtained PZT film showed high (001)-oriented growth with *F*(001) = 0.92 without any micro-cracks. The RLNO was only uniaxial-oriented grown at the top part of the RLNO amorphous precursor layer. The orientedly grown and amorphous phases of RLNO would have two important roles for this multilayered film formation: (1) triggering orientation growth of the PZT film at the top, and (2) the stress relaxation of the underneath BTO layer to suppress the micro-crack formation. This is the first time that PZT films have been crystallized directly on flexible substrates by the combined process of photocrystallization and CSD, which is a cost-effective and highly on-demand process. The “print-on-demand” method will open new pathways for the fabrication of self-powered devices.
